# Keratin 12 mRNA expression could serve as an early corneal marker for limbal explant cultures

**DOI:** 10.1007/s10616-020-00373-z

**Published:** 2020-02-03

**Authors:** Lei Shi, Tanja Stachon, Barbara Käsmann-Kellner, Berthold Seitz, Nóra Szentmáry, Lorenz Latta

**Affiliations:** 1 Department of Ophthalmology, Saarland University Medical Center, Kirrberger Str. 100, 66424 Homburg, Saar Germany; 2 The First Affiliated Hospital of USTC, Division of Life Science and Medicine, University of Science and Technology of China, Hefei, Anhui People’s Republic of China; 3 Department of Ophthalmology, Semmelweis University, Budapest, Hungary

**Keywords:** Limbal explant, Limbus, Corneal epithelial progenitors, Differentiation, qPCR, KRT12

## Abstract

This investigation aimed to identify early corneal marker and conjunctival epithelial differentiation through transcriptional analysis of limbal explant cultures and study early differentiation patterns of known corneal and conjunctival differentiation markers. 2 mm punch biopsies of limbal region were obtained from 6 donors of the Lions Cornea Bank Saar-Lorloux/Trier-Westpfalz. Limbal explants were dissected into corneal and conjunctival biopsy sections. Biopsies were placed with epithelial side down into 12 Wells. As soon as the outgrowing cells had reached confluence, they were harvested. mRNA expression of corneal differentiation markers KRT12, KRT3, DSG1, PAX6, ADH7 and ALDH1A1, conjunctival markers KRT19, KRT13 and stem cell marker ABCG2 were measured via qPCR. KRT12 and PAX6 protein expressions were evaluated using Western Blot. Results suggested that KRT12 mRNA expression was significantly higher in outgrowing cells from the corneal side of the biopsies as in those from the conjunctival side (p = 0.0043). There was no significant difference in mRNA expression of other analyzed markers comparing with marker expression of outgrown cells from both limbal biopsies (p > 0.13). KRT12 and PAX6 Western Blot analysis showed no difference in cells harvested from both sides. In conclusion, KRT12 mRNA might be a marker to measure corneal origin of cells from limbal biopsies with unknown composition of corneal and conjunctival progenitor cells. KRT3, DSG1, PAX6, ADH7, ALDH1A1, KRT19, KRT13 and ABCG2 mRNA as well as KRT12 and PAX6 protein expression could not contribute to differentiate corneal from conjunctival cell identity from limbal biopsies.

**Electronic supplementary material:**

The online version of this article (10.1007/s10616-020-00373-z) contains supplementary material, which is available to authorized users.

## Introduction

Since the proof of concept manifested that limbal stem cell transplantation can be beneficial for patients with limbal stem cell deficiency (Pellegrini et al. 1997), a lot of effort has been undertaken to define the quality of transplanted cells as well as optimizing cell culture. The first concept on limbal stem cells has been developed through the knowledge that epithelial stem cells are able to form holo-, mero- and paraclones (Barrandon and Green 1987). The clonal analysis on feeder layer was first successfully proven by Pellegrini et al. (2001) and they described that holoclones correlate with p63α expression (stem cell marker) (Pellegrini et al. 2001; Di Iorio et al. 2005). Later on they have measured p63α expression to control the quality of the cell cultures (Di Iorio et al. 2006) and have linked its expression to clinical outcome in patients (Rama et al. 2010). The limbal stem cell niche and the corneal and conjunctival epithelium have been well characterized in various extensive studies (Schlotzer-Schrehardt and Kruse 2005; Schlotzer-Schrehardt et al. 2007; Nakatsu et al. 2011, 2013; Ramirez-Miranda et al. 2011; Zhou et al. 2006; Figueira et al. 2007; Kulkarni et al. 2010; Takács et al. 2011; Chen et al. 2004). However, there is some contradiction in these studies concerning gene expression, which is likely due to varying reactivities of the antibodies utilized. KRT3, KRT12, DSG1, ADH7 and ALDH1A1 as corneal expression markers and KRT13 and KRT19 as conjunctival markers have been described (Schlotzer-Schrehardt and Kruse 2005; Nakatsu et al. 2011; Davis et al. 2003; Kitazawa et al. 2017; Turner et al. 2007). Especially surface markers are of interests with the aim to enrich stem cells.

There is growing evidence to demonstrate that niche cells function importantly in stem cell homeostasis (Polisetti et al. 2016). The cell niche in vitro is often “simulated” by presence of a feeder cell layer or by amniotic membrane as scaffold. The exact site of the biopsy as “corneal” or “conjunctival” site must be considered. Another issue is that stem cell markers are not specific for corneal or conjunctival progenitors as limbus is the transition zone for both epithelia (Ramos et al. 2015). Also, it is not clear if antibodies which can discriminate corneal and conjunctival phenotype in tissues are suitable for validating differentiation in cell culture. Taking everything into consideration, we need to better understand the early differentiation process and cell fate decision of cornea epithelial cells derived from limbus. The aim of the investigation was to utilize qPCR for quality grading or understanding of the early differentiation process of limbal epithelial cell culture. In order to achieve this, we analyzed limbus explant cultures with established corneal and conjunctival differentiation markers on mRNA level and looked for distribution and correlation of theses markers across the limbus. These samples could provide a good model to study this purpose as the samples (split biopsies) should provide isogenic cells with differences in cell lineage or differentiation stage mixture, and are very close to cells that used clinically.

## Materials and methods

### Ethical considerations

All experiments were conducted according to the tenets of the Declaration of Helsinki. The use of corneal scleral donor rims the research project was approved by the Ethics Committee of the Saarland (Number 226/15).

### Limbal epithelial explant culture

Preparation of limbal biopsies is shown in Supplementary Fig. 1. Limbal tissue had been removed using a 2 mm Punch (Acuderm inc., Fort FL, USA), forceps and spring scissors from donor tissue of the Lions Cornea Bank Saar-Lorlux/Trier-Westpfalz (Homburg/Saar, Germany). The average age of corneal donors was 67.5 years ranging from 31 to 85 years. Post mortem time ranged up to 18 h. Six to eight biopsies per donor rim were excised and dissected with surgical scalpel into conjunctival and corneal parts, which were then placed with epithelial side down into 12 well plates. Thereafter, KSFM medium was carefully added avoiding explant detachment. Outgrowth was observed from our explants and after growing cells reached confluence, limbal explants were removed from the corneal limbal epithelial cultures (Cor-LEC) and from the conjunctival limbal epithelial cultures (Conj-LEC). Cells derived from the same donor but cultured in separate explant cultures were pooled later, which were analyzed together.

### RNA and protein extraction and cDNA synthesis

Cells from explant cultures were lysed and processed with an RNA/DNA/Protein isolation kit (Isolate II, Bioline, London, UK) according to the manufacturer’s instructions. RNA quantity was determined using UV/VIS spectrophotometry (Nanodrop 1000, PeqLab, Erlangen, Germany). Protein concentration was analyzed with a Bradford Kit (Merck, Darmstadt, Germany). One Taq RT-PCR Kit (New England Biolabs INC, Frankfurt, Germany) was used to convert total RNA to cDNA with M-MulV Enzyme Mix and oligo dT primers. We used 500 ng of total RNA for one cDNA reaction.

### Quantitative PCR (qPCR) analysis

For qPCR measurement, primer sets (Table 1) were mixed with ACEq DNA SYBR Green Mix (Vazyme). Samples were run in 12.5 µl volume using 0.5 µl cDNA and primer concentration according to the standard procedure. The qPCR experiments (n = 6) were carried out in 96-well plates as duplicates, which were measured with a PCR Thermocycler CFX Connect (BioRad Laboratories München, Germany). The amplification conditions were 95 °C for 10 s, 60 °C for 30 s and a total of 40 cycles. An annealing temperature of 64 °C was used for KRT13. The Cq values were analyzed from BioRad CFX Manager Software 3.1. Fold differences were calculated using the ΔΔCq method. For comparison of mRNA expressions for Cor-LEC and Conj-LEC, the Cor-LEC preparation of each donor was used for normalization. In further analysis, the correlation of different markers and variation in expression among the samples is obtained. The means of Cor-LEC ΔCq of all preparations was used for normalization (2^ΔΔCq^). A log scale for fold difference was applied to better visualize the variability among the different samples. Additionally, expression of Conj-LEC was compared regarding mean ΔCq of all Conj-LEC samples. PAX6 splice ratio analysis with TaqMan assays see (Table 1 Part 2). A Run was performed on QuantStudio5 with TaqMan advances master mix according tomanufacturer’s instructions. Primers and Probes were purchased at MWG Eurofins. Expression was calculated using the ΔΔCq method and PAX6a signal was used as reference.

**Table 1:** Qiagen QuantiTect Primer pairs used for qPCR (Part1) and for TaqMan assay (Part2) (BHQ: black whole quencher, MBG: minor grove binding)

Part 1		
Targeted mRNA transcripts	Cat. no	Amplicon size (bp)
ABCG2: NM_004827, NM_001257386	QT00073206	114
ADH7: NM_000673, NM_001166504	QT00000217	85
ALDH1A1: NM_000689	QT00013286	97
DSG1: NM_001942	QT00001617	96
KRT13: NM_002274 NM_153490	QT00068747	60
KRT12: NM_000223	QT00011949	104
KRT19: NM_002276	QT00081137	117
KRT3: NM_057088	QT00050365	118
PAX6: NM_000280, NM_001127612, NM_001604, NM_001258462, NM_001258463, NM_001258464, NM_001258465	QT00071169	113
TBP: NM_001172085, NM_003194	QT00000721	132

Part 2		
TaqMan primer and probe sequences primer 5′ → 3′ (targeted mRNA transcripts)	Sequence/dye	Amplicon size (bp)
PAX6 Fw		
(NM_000280.4, NM_001604.5, NM_001127612.1, NM_001258462.1	GGCCGTGCGACATTTCC	66/108
PAX6 Rev		
NM_001258463.1, NM_001258464.1, NM_001258465.1, NM_001310158.1, NM_001310159.1, 10 NM_001310160.1, NM_001310161.1)	ACCTGCCCAGAATTTTACTCACA	66/108
PAX6a_Probe (NM_000280.4, NM_001127612.1, NM_001258464.1, NM_001258465.1 NM_001310159.1)	AATTCTGCAGGTGTCCAA/FAM_BHQ_MBG	–
PAX6b_Probe (NM_001604.5, NM_001258462.1, NM_001258463.1, NM_001310158.1, NM_001310160.1, NM_001310161.1)	CCCATGCAGATGCAA/YAKIMA_BHQ_MBG	–

### Western blot

Total protein (20 µg) from each preparation was denatured and separated on a precast 4–12% NuPageTM Bis–Tris SDS Gel (Invitrogen, Waltham, MA, USA). Separated proteins were transferred onto a nitrocellulose membrane and probed with antibodies against mouse PAX6 (Santa Cruz, sc-32766, 1:200 Santa Cruz, CA, USA) and KRT12 (Santa Cruz, Sc-515882, 1:200, Santa Cruz, CA, USA). The western blot was reprobed with mouse α-ACTB antibody (Abcam, ab8227 1:5000, Cambridge, UK) as a loading control. Antibodies were diluted with a WesternFroxx Kit (BioFroxx GmbH, Einhausen, Germany). For detection, a western lightning chemiluminescence reagent, Plus ECL, was used (Perkin Elmer Life Sciences, Waltham, MA, USA). Images were acquired with a LAS 4000 System (Fuji Film, Tokio, Japan).

### Data analysis and statistics

Data analysis was completed with Excel 2016 (Microsoft Redmond, WA, USA). Graphs and statistical analysis on ΔCq were processed with GraphPad Prism 7.04 (GraphPad Software, Inc, La Jolla, CA, USA). Statistical analysis was performed using a Mann–Whitney Test comparing ΔCq expression values of Cor-LEC with Conj-LEC. P values below 0.05 were considered as statistically significant. Additionally, Spearman correlation (two sided) analysis of ΔCq was performed to identify co-regulated differentiation markers. The resulting correlation matrix file was then imported via metscape plug-in (Basu et al. 2017) for Cytoscape (Shannon et al. 2003), using a cutoff of 0.5.

## Results

**Fig. 1:**
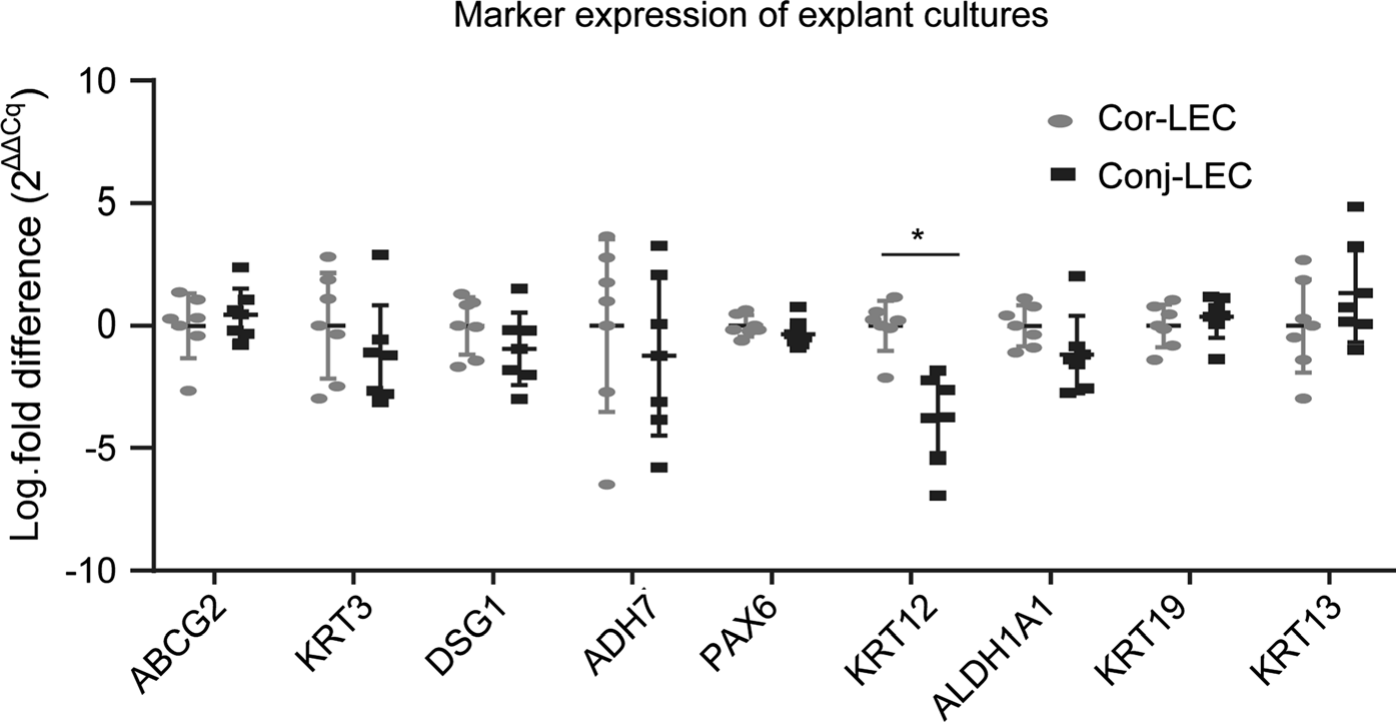
The ΔΔCq expression fold change differentiation and stem cell markers between corneal limbal explant cultures (Cor-LEC) and conjunctival limbal explant cultures (Conj-LEC). Expression was normalized to Cor-LEC samples, respectively and log scaled. The mean expression of corneal markers DSG1, KRT12, KRT3 is reduced in Conj-LEC. Conjunctival marker KRT13 and KRT19 were elevated in Conj-LEC samples. KRT13, ABCG2 ADH7 and ALDH1A1 show very high deviation in expression across different samples. For statistical analysis ΔCq values of each expression marker were compared between Cor-LEC and Conj-LEC using a Mann–Whitney test. KRT12 is significantly reduced in Conj-LEC Samples. *p < 0.05. (Cor-LEC: Corneal limbal explant culture, Conj-LEC: conjunctival limbal explant culture)

Figure 1 shows relative quantification of marker expression for Cor-LEC cultures vs. Conj-LEC cultures. Mean expression fold differences in Conj-LEC samples were between 0.5 to fourfold expression change with high deviation across the samples, as seen on high variability across the preparations for several differentiation markers, especially KRT13, KRT3 and stem cell marker ABCG2. KRT12 was the only marker significantly reduced (mean fold difference of 0.08; p = 0.0043) showing similar relative expression between the samples for Cor-LEC and Conj-LEC (Supplementary Fig. 1). In order to show how expression was distributed across the samples, we displayed the data normalized to the mean of all Cor-LEC samples (Supplementary Figs. 2A, 3A, 4A). With this graphical presentation correlation analysis results can be better compared to relative expression levels (Supplementary Figs. 2B, 3B, 4B). Three corneal differentiation markers and putative stem cell marker ABCG2 are displayed in Supplementary Fig. 2. Preparation 4 and 5 showed highest expression of ACBG2 marker between Cor-LEC and Conj-LEC. For ABCG2 there was no tendency of higher expression in either Cor-LEC or Conj-LEC samples. The corneal differentiation markers KRT3, DGS1 and ADH7 were very little expressed in preparation 4–5 regardless from which position of the limbal explant cells they derived from (Cor-LEC or Conj-LEC). For preparation 6 there was much higher expression of KRT3, DSG1 and AHD7 in Cor-LEC compared to Conj-LEC sample. The markers ABCG2, KRT3, DSG1 and ADH7 (Supplementary Fig. 2A) showed a similar profile in expression across different preparations which was supported by correlation analysis (Supplementary Fig. 2B). ABCG2 negativly correlates to differentiation marker expression of KRT3, DSG1 and ADH7.

Another set of markers is displayed in Supplementary Fig. 3 namely PAX6, ALDH1A and KRT12. Keratin12 has been reported by others to be regulated by PAX6 (Chaloin-Dufau et al. 1990). PAX6 shows a slight reduction of PAX6 expression levels in Conj-LEC, except preparation 2. KRT12 was highly reduced in all Conj-LEC samples within all preparations. ALDH1A1 showed a similar expression pattern of PAX6 indicated by higher correlation of expression of ALDH1A1 and PAX6 markers compared to KRT12 and PAX6 (Supplementary Fig. 3B).

**Fig. 2:**
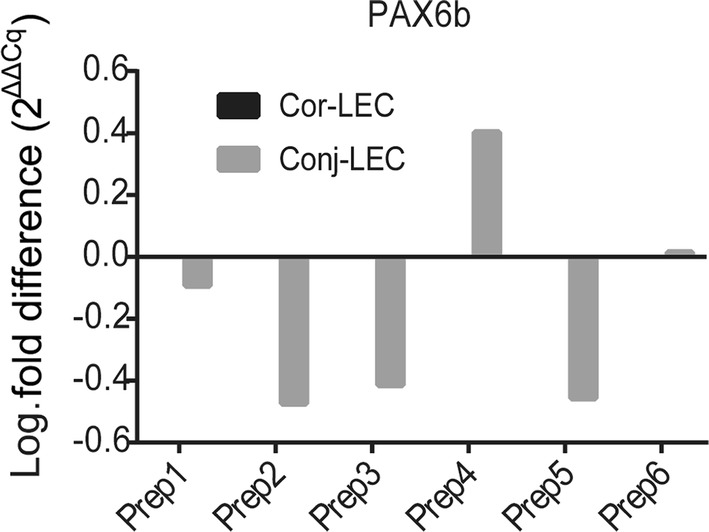
PAX6b expression normalized to PAX6a expression. In Conjunctiva there is no significant change of PAX6 splice variant ratio between Cor-LEC and Conj-LEC cell cultures. Ratios vary between samples. (Cor-LEC: Corneal limbal explant culture, Conj-LEC: conjunctival limbal explant culture). Y axis is log-scaled fold difference

In Supplementary Fig. 3C, Western Blot of PAX6 and KRT12 is shown. We could not detect a difference in protein expression between Cor-LEC and Conj-LEC or across different preparations. Since PAX6 does not show obvious expression variations which could explain the huge differences in KRT12 expression, we checked for differently expressed PAX6 splice isoforms PAX6a and PAX6b which were already reported to differentially regulate KRT12 and KRT3. But there was no difference of ratio of PAX6 splice variants (Fig. 2).

The means of conjunctival markers KRT19 (fold difference 1.3) and KRT13 (fold difference 2.5) are higher expressed Conj-LEC. KRT19 showed less variation in expression across different preparations. There was no correlation between conjunctival markers KRT19 and KRT13 markers using threshold of 0.5 (Supplementary Fig. 4B). We did not identify any correlation of differentiation markers to age, sex, post mortem time or culture duration.

## Discussion

Obtaining corneal and conjunctival explant culture by splitting a 2 mm limbal biopsy in the middle those results show the potential in sensitivity of qPCR. Corneal limbal explant cultures (Cor-LEC) showed higher corneal marker expression compared to conjunctival limbal explant cultures (Conj-LEC). ABCG2 as a putative stem cell marker showed no preference in expression to Cor-LEC vs. Conj-LEC in our preparations. However, the corneal and conjunctival identity can only be detected in Cor-LEC if its expression is compared to its corresponding Conj-LEC from the same donor. We observed few exceptions where corneal markers were higher expressed in Conj-LEC and conjunctival markers lower expressed in Conj-LEC. Possible reason is that due to the high fluctuations in marker expression across different preparations, it is difficult or impossible to rule out expression thresholds for general quality measurements. Within the common corneal differentiation markers such like KRT3, DSG1 and ADH7, the expression changes between the different samples are higher than the expression differences between Cor-LEC vs. Conj-LEC from the same preparation. Nevertheless, KRT12 protein expression did not change parallel to the rather dramatic difference in mRNA expression between both groups. Therefore, KRT12 protein might not be used as an early differentiation marker for grading limbal epithelial cell cultures. HOLOCLAR^®^ uses KRT3 to distinguish limbal biopsies from biopsies that accidentally exhibited too big a proportion of conjunctival progenitors. It has been described that KRT3 and KRT12 protein expression is initiated variously in different organisms or different conditions (e.g. developmental stage, cell culture) (Chaloin-Dufau et al. 1990). This might explain the difference in expression patterns of the differentiation markers KRT3, DSG1, ADH7 and the marker KRT12. Understanding regulators and controlling these differentiation markers might help to optimize culture conditions and allow quality control. In 3T3 fibroblast cell line of non-corneal origin, regulation through PAX6 expression did not seem to be sufficient in order to regulate keratin expression since keratin genes are not among identified differential regulated genes (Kiselev et al. 2012). There were no significant differences in PAX6a/PAX6b mRNA ratio comparing Cor-LEC vs Conj-LEC samples from the same donors. Further critical parameters like stratification and relating signaling pathways, which depend on calcium and cell–cell interaction have to be studied in the future (Leiper et al. 2006; Harmon et al. 2013). Factors influencing differentiation could also be asymmetrically distributed in limbal stroma of explant cultures.

In summary, KRT12 mRNA may be a marker to support differentiation between conjunctival and corneal limbal cells but with moderate variations between individuals. KRT3, DSG1, PAX6, ADH7, ALDH1A1, KRT19, KRT13, ABCG2 mRNA as well as KRT12 and PAX6 protein expression does not help to differentiate corneal and conjunctival limbal stem cells grown from limbal biopsy. Much more effort should be spent analyzing corneal and conjunctival cell fate in future.

## Electronic supplementary material

Below is the link to the electronic supplementary material.

Supplementary material 1 (TIFF 2812 kb). **Fig. S1.** Schematic view of limbus and limbus biopsies taken from corneoscleral donor rims. Biopsies were split and put with epithelial down in 12 Well plates. After reaching confluence explants were removed and cells were pooled and lysed. (Cor-LE: Corneal limbal explant, Conj-LE: conjunctival limbal explant; Cor-LEC: Corneal limbal explant culture, Conj-LEC: conjunctival limbal explant culture).

Supplementary material 2 (TIFF 882 kb). **Fig. S2. A)** Comparison fold difference expression between different preparations. Values were normalized to mean ΔCq of Cor-LEC and log scaled. Preparation 2 shows higher expression of corneal markers DSG1 und KRT3 in Conj-LEC samples. Preparation 4 and 5 show very low expression of corneal differentiation markers compared to the mean of all samples. Preparation 6 shows high expression of corneal differentiation marker in Cor-LEC and huge difference between Cor-LEC and Conj-LEC samples compared to other preparations. **B)** From all samples, a correlation analysis was performed to identify co-regulations of stem cell and differentiation markers using Spearman correlation of ΔCq values. ACBG2 correlates negatively (blue color) with corneal differentiation markers KRT3, DSG1 and ADH7. Thickness of lines indicated correlation) Red circles refer to figures showing expression fold differences for single samples normalized to mean of all samples. (Cor-LEC: Corneal limbal explant culture, Conj-LEC: conjunctival limbal explant culture).

Supplementary material 3 (TIFF 961 kb). **Fig. S3. A)** Comparison of fold difference expression between different preparations. Values were normalized to mean ΔCq of Cor-LEC and log scaled. **B)** Correlation analysis of shown markers. **C)** Western blot of four individual preparations showing KRT12 and PAX6 expression, respectively. β-Actin (ACTB) was used as loading control. (Cor-LEC: Corneal limbal explant culture, Conj-LEC: conjunctival limbal explant culture).

Supplementary material 4 (TIFF 711 kb). **Fig. S4. A)** Comparison of fold difference expression between different preparations. Values were normalized to mean ΔCq of Cor-LEC and log scaled. **B)** Correlation analysis of shown markers. (Cor-LEC: Corneal limbal explant culture, Conj-LEC: conjunctival limbal explant culture).
