# Supplementary figures and images for: Keratin 12 mRNA expression could serve as an early corneal marker for limbal explant cultures
Source: Cytotechnology. 2020 Feb 3;72(2):239–45. doi: 10.1007/s10616-020-00373-z (PMC7192984; doi:10.1007/s10616-020-00373-z)

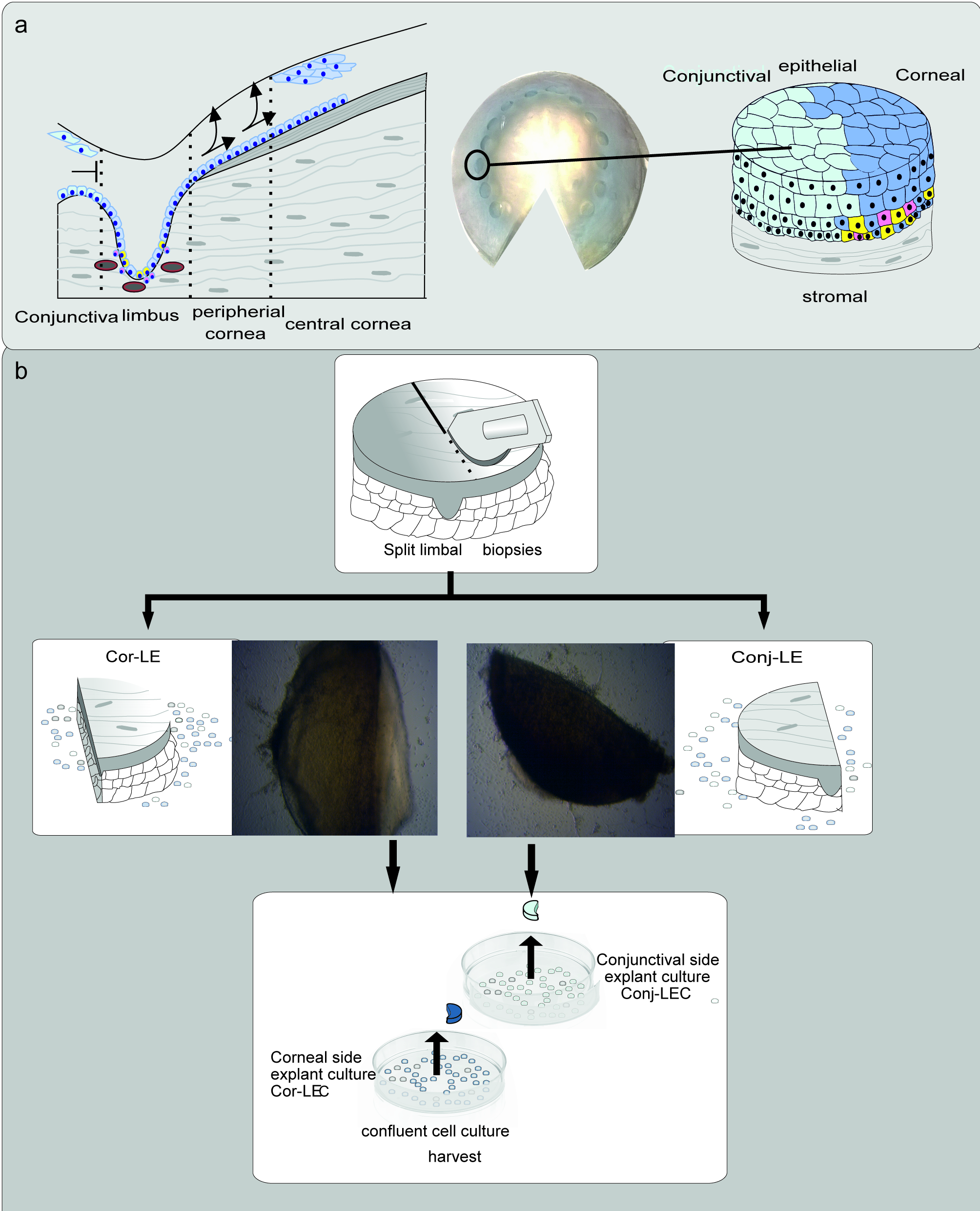

Supplement: Supplementary file 1 — Supplementary material 1 (TIFF 2812 kb). Fig. S1. Schematic view of limbus and limbus biopsies taken from corneoscleral donor rims. Biopsies were split and put with epithelial down in 12 Well plates. After reaching confluence explants were removed and cells were pooled and lysed. (Cor-LE: Corneal limbal explant, Conj-LE: conjunctival limbal explant; Cor-LEC: Corneal limbal explant culture, Conj-LEC: conjunctival limbal explant culture). [file 10616_2020_373_MOESM1_ESM.tif]

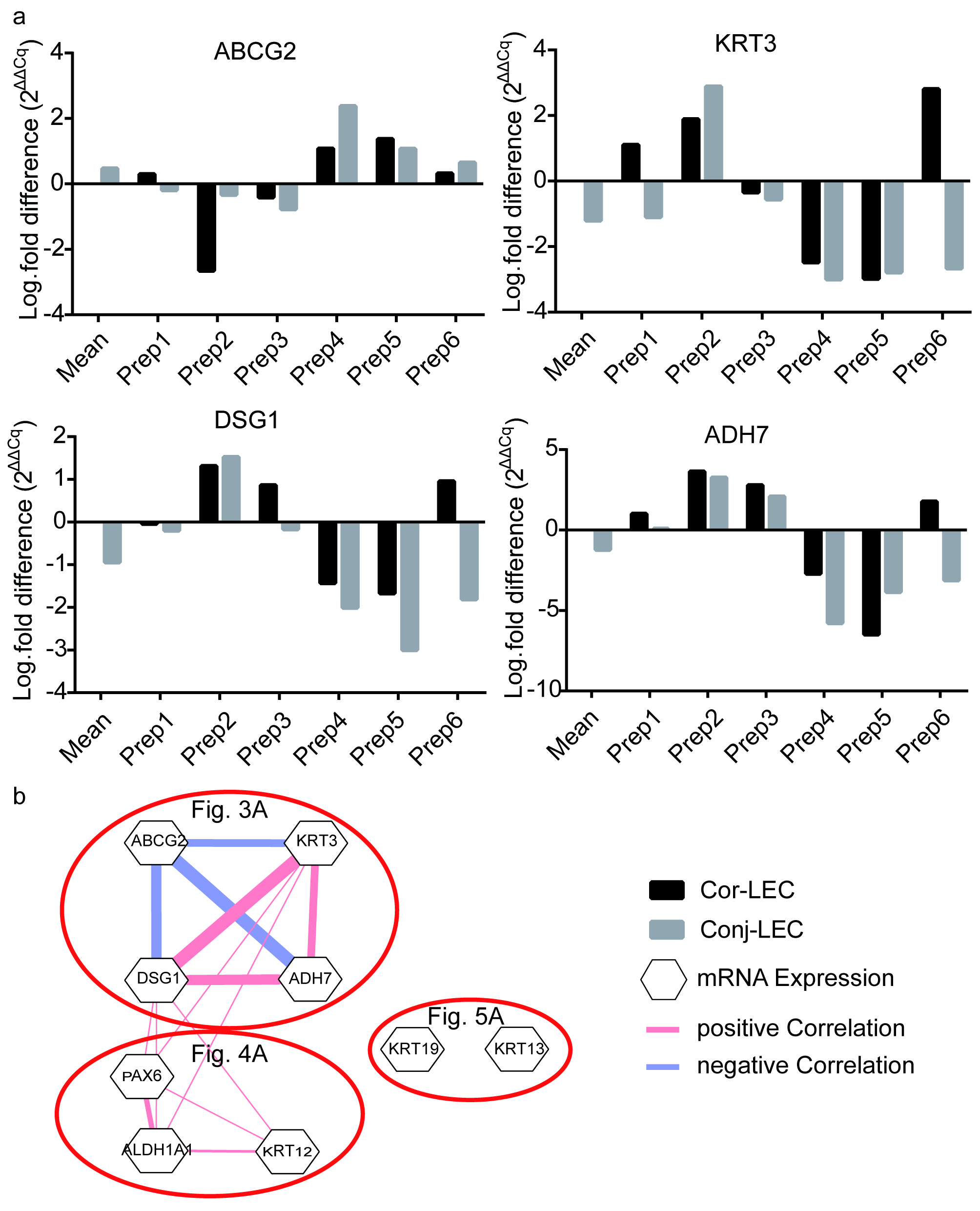

Supplement: Supplementary file 2 — Supplementary material 2 (TIFF 882 kb). Fig. S2. A) Comparison fold difference expression between different preparations. Values were normalized to mean ΔCq of Cor-LEC and log scaled. Preparation 2 shows higher expression of corneal markers DSG1 und KRT3 in Conj-LEC samples. Preparation 4 and 5 show very low expression of corneal differentiation markers compared to the mean of all samples. Preparation 6 shows high expression of corneal differentiation marker in Cor-LEC and huge difference between Cor-LEC and Conj-LEC samples compared to other preparations. B) From all samples, a correlation analysis was performed to identify co-regulations of stem cell and differentiation markers using Spearman correlation of ΔCq values. ACBG2 correlates negatively (blue color) with corneal differentiation markers KRT3, DSG1 and ADH7. Thickness of lines indicated correlation) Red circles refer to figures showing expression fold differences for single samples normalized to mean of all samples. (Cor-LEC: Corneal limbal explant culture, Conj-LEC: conjunctival limbal explant culture). [file 10616_2020_373_MOESM2_ESM.tif]

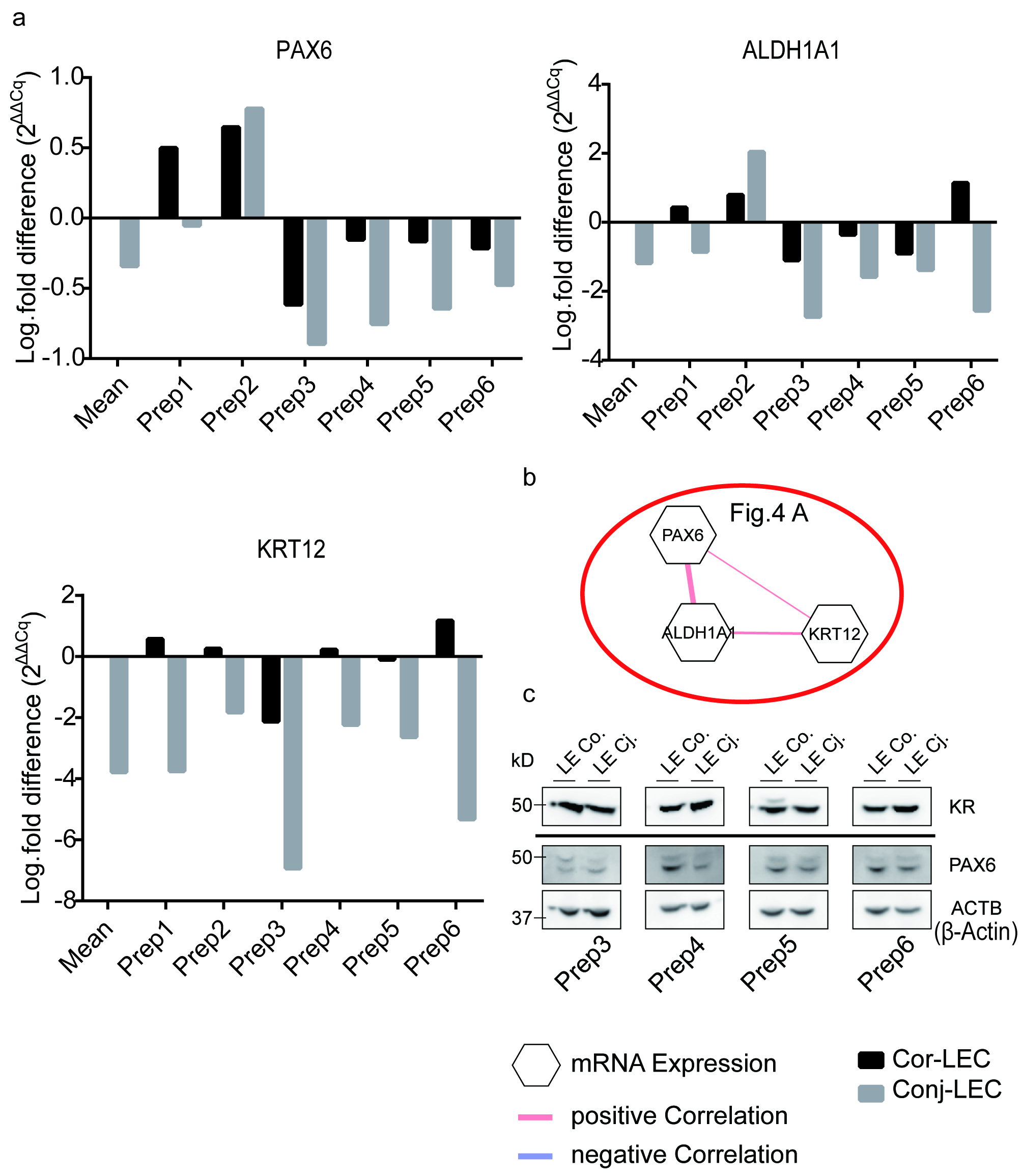

Supplement: Supplementary file 3 — Supplementary material 3 (TIFF 961 kb). Fig. S3. A) Comparison of fold difference expression between different preparations. Values were normalized to mean ΔCq of Cor-LEC and log scaled. B) Correlation analysis of shown markers. C) Western blot of four individual preparations showing KRT12 and PAX6 expression, respectively. β-Actin (ACTB) was used as loading control. (Cor-LEC: Corneal limbal explant culture, Conj-LEC: conjunctival limbal explant culture). [file 10616_2020_373_MOESM3_ESM.tif]

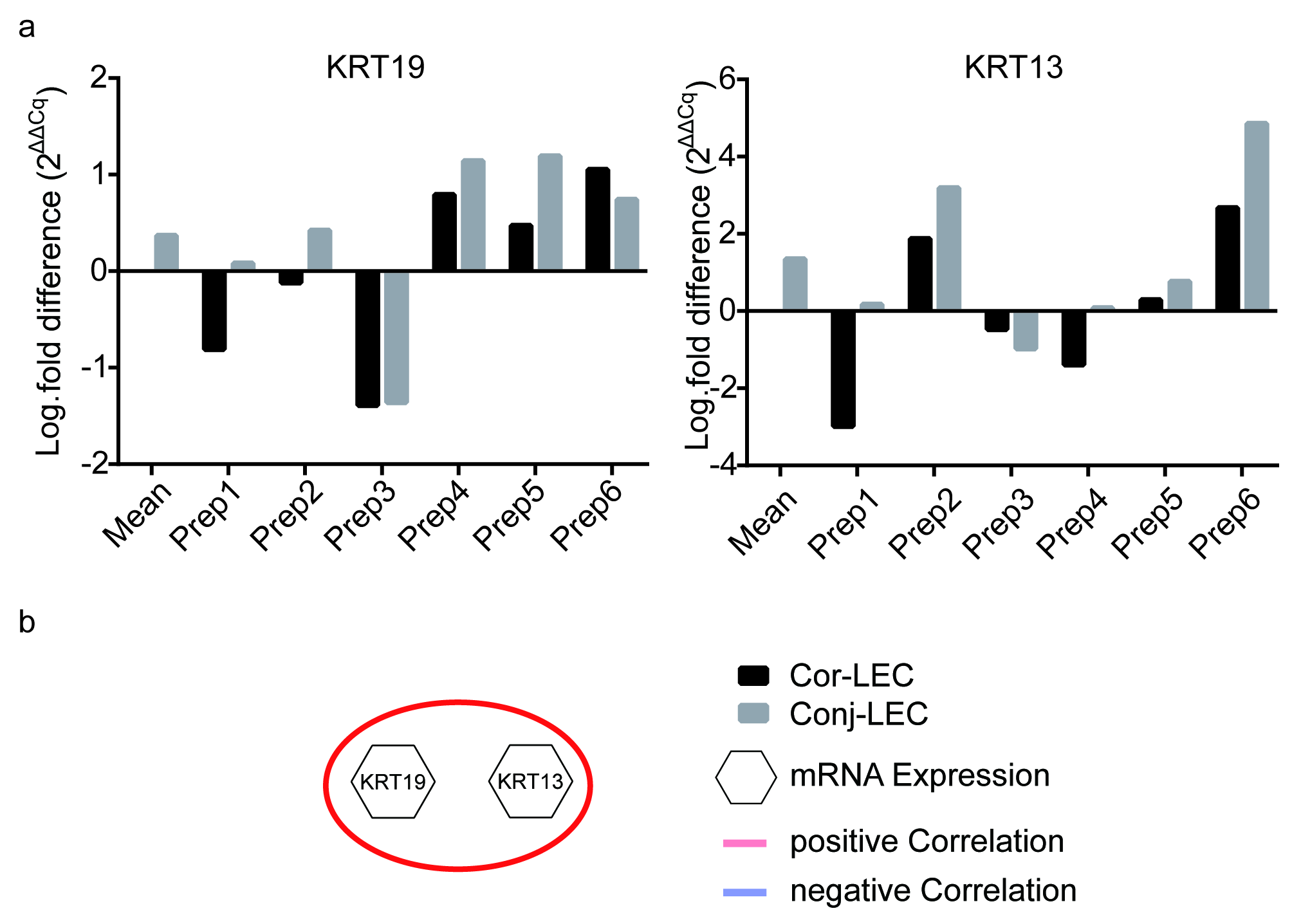

Supplement: Supplementary file 4 — Supplementary material 4 (TIFF 711 kb). Fig. S4. A) Comparison of fold difference expression between different preparations. Values were normalized to mean ΔCq of Cor-LEC and log scaled. B) Correlation analysis of shown markers. (Cor-LEC: Corneal limbal explant culture, Conj-LEC: conjunctival limbal explant culture). [file 10616_2020_373_MOESM4_ESM.tif]
